# An Endogenous Vitamin K-Dependent Mechanism Regulates Cell Proliferation in the Brain Subventricular Stem Cell Niche

**DOI:** 10.1002/stem.1045

**Published:** 2012-01-30

**Authors:** Aurore Gely-Pernot, Valérie Coronas, Thomas Harnois, Laetitia Prestoz, Nathalie Mandairon, Anne Didier, Jean Marc Berjeaud, Arnaud Monvoisin, Nicolas Bourmeyster, Pablo García De Frutos, Michel Philippe, Omar Benzakour

**Affiliations:** a Institut de Physiologie et Biologie Cellulaires, CNRS-UMR 6187 Université de PoitiersPoitiers, France; b CHU de PoitiersRue de la Miletrie, Poitiers, France; c Lyon Neuroscience Research Center, Université Lyon1 Lyon cedexFrance; d Equipe de Microbiologie, Université de PoitiersCNRS 5292, Inserm 1028, Poitiers, France; e Instituto de Investigaciones Biomédicas de Barcelona(CSIC) Barcelona, CNRS-UMR 6008, Spain

**Keywords:** Neural stem cell, vitamin K-dependent proteins, brain subventricular zone, protein S, Gas6

## Abstract

Neural stem cells (NSC) persist in the adult mammalian brain, within the subventricular zone (SVZ). The endogenous mechanisms underpinning SVZ stem and progenitor cell proliferation are not fully elucidated. Vitamin K-dependent proteins (VKDPs) are mainly secreted factors that were initially discovered as major regulators of blood coagulation. Warfarin ((S(−)-3-acetonylbenzyl)-4-hydroxycoumarin)), a widespread anticoagulant, is a vitamin K antagonist that inhibits the production of functional VKDP. We demonstrate that the suppression of functional VKDPs production, in vitro, by exposure of SVZ cell cultures to warfarin or, in vivo, by its intracerebroventricular injection to mice, leads to a substantial increase in SVZ cell proliferation. We identify the anticoagulant factors, protein S and its structural homolog Gas6, as the two only VKDPs produced by SVZ cells and describe the expression and activation pattern of their Tyro3, Axl, and Mer tyrosine kinase receptors. Both in vitro and in vivo loss of function studies consisting in either Gas6 gene invalidation or in endogenous protein S neutralization, provided evidence for an important novel regulatory role of these two VKDPs in the SVZ neurogenic niche. Specifically, we show that while a loss of Gas6 leads to a reduction in the numbers of stem-like cells and in olfactory bulb neurogenesis, endogenous protein S inhibits SVZ cell proliferation. Our study opens up new perspectives for investigating further the role of vitamin K, VKDPs, and anticoagulants in NSC biology in health and disease. Stem Cells *2012; 30:719–731*

## INTRODUCTION

In the adult mammalian organism, tissue-specific stem cells replenish throughout life organs by replacing lost cells [1]. The main reservoir of stem cells in the brain is the subventricular zone (SVZ) bordering the lateral ventricles [2–4]. Under physiological conditions, SVZ neural stem cells (NSCs) provide a continuous supply of neuroblasts that migrate toward the olfactory bulb, where they ensure turnover of interneurons, and also give birth to glial cells that disperse in the white matter of diverse brain regions [2–4]. Brain injuries including neurodegenerative diseases or ischemia are believed to mobilize NSCs, providing thereby neuroblasts to injured brain areas that replace part of dying neurons [5–8]. Cues derived from NSC and from the surrounding cells of the stem cell niche as well as signals of remote brain areas regulate NSC proliferation and differentiation of their progenies [9–11]. Although much attention has been devoted to identifying exogenous factors that promote neural stem and progenitor cell proliferation [9–13], little is known of the endogenous regulatory mechanisms, particularly the inhibitory ones that may constitutively contribute to regulating cell proliferation within the SVZ stem cell niche or within other stem cell niches.

Vitamin K-dependent proteins (VKDPs) are a family of proteins characterized by vitamin K-dependent post-translational modifications of some glutamyl residues to γ-carboxyglutamic acid residues [14, 15]. This reaction is catalyzed by the γ-glutamyl carboxylase and requires vitamin K in its reduced form as a cofactor [14, 15]. During the gamma carboxylation process, vitamin K is oxidized in vitamin K 2,3,-epoxide, which is recycled back to its reduced form by a vitamin K epoxide reductase enzyme system [14, 15]. Warfarin ((S(−)-3-acetonylbenzyl)-4-hydroxycoumarin)) and its derivatives inhibit the vitamin K epoxide reductase, blocking thereby the γ-carboxylation reaction [14, 15]. VKDPs synthesized in the presence of warfarin are under-γ-carboxylated and are either not secreted but degraded intracellularly or have impaired biological activities [14–17]. As VKDPs are mainly secreted factors most of which regulate blood coagulation [15–17], the specificity and efficiency of warfarin in inhibiting the γ-carboxylation reaction have led to its widespread use in oral anticoagulant therapy [14]. Processes regulated by the VKDPs are referred to as vitamin K-dependent mechanisms and warfarin treatment as anti-vitamin K therapy [14–18].

Based on preliminary but consistent observations that the vitamin K antagonist, warfarin, induces proliferation of SVZ cell cultures, we hypothesized that endogenous VKDPs produced by SVZ cells may represent an SVZ endogenous regulatory mechanism. Therefore, we undertook experiments to elucidate this hypothetical mechanism. We identify the anticoagulant factors, protein S and its structural homolog Gas6, as the two only VKDPs produced by SVZ cells. Protein S and its structural homolog Gas6, in addition to their role in blood coagulation, interact with their tyrosine kinase receptors Tyro3, Axl, and Mer (TAM) and exert cellular effects [19–26]. Using multiple approaches, we analyzed the effects of both Gas6 and protein S on SVZ cells and established, using loss of function studies consisting in either Gas6 gene invalidation or in endogenous protein S neutralization, an important novel regulatory role of these two VKDPs in the SVZ germinative niche.

## MATERIALS AND METHODS

### Primary Cultures of SVZ Cells

Animals were handled and all experimental procedures were carried out in accordance with the guidelines of the French Agriculture and Forestry Ministry (decree 87849) and of the European Communities Council Directive (86/609/EEC). Brain SVZ cells were derived from 1- to 3-day-old rats (R. Janvier) or from either adult Wistar rats or C57BL/6J mice and from newborn C57Bl/6J wild type or Gas6 knockout (Gas6^−/−^) mice. The original Gas6^−/−^ mouse line [27] was back-crossed into a C57Bl/6J background for more than 10 generations, yielding congenic C57Bl/6J wild type and Gas6^−/−^mice. SVZ cell cultures were performed as previously described [13] in minimal essential medium (for newborn animals) or neurobasal (for adult animals) media supplemented with B27 and with 20 ng ml^−1^ epidermal growth factor (EGF). For all in vitro assays, data were obtained from at least three independent experiments each in quadruplicates.

### Preparation of SVZ Cell Culture Conditioned Media

SVZ neurosphere cultures were allowed to develop for 5 days in serum-free medium (SFM) supplemented with 20 ng ml^−1^ EGF. Cell cultures were then placed for 5 additional days in either SFM (control) or SFM supplemented with 1 μg ml^−1^ warfarin (Sigma) or 10 μg ml^−1^ vitamin K1 (Roche Diagnostic, http://www.rochediagnostics.fr). SVZ cell culture conditioned media (supernatant) were collected and then concentrated by ultrafiltration for 2 hours at 3,500 g using YM-3 Centricon filter device (Millipore). The upper fractions (molecular weight [MW] >3,000 Da) were collected and immediately used for SVZ cell culture growth assays. HPLC analysis was used for monitoring effective removal of warfarin or vitamin K1 from SVZ conditioned media following the ultrafiltration procedure (Supporting Information Fig. 3)

### Recombinant Murine Gas6 (rmGas6) Preparation

HEK293 cells stably transfected with the pcDNA3.1 expressing vector encoding the full-length murine Gas6 were kindly given by Dr M. Hall [28]. Cell clones secreting high levels of rmGas6 were propagated in complete medium with added vitamin K1 (10 μg/ml). rmGas6 was purified from conditioned medium using the barium citrate precipitation method as described [29].

### SVZ Neurosphere Forming, Self-Renewal, and Cell Culture Growth Assays

For SVZ neurosphere forming and self-renewal assays, SVZ cells derived from wild type or Gas6^−/−^ mice were seeded at 10 cells per microliter in 24 well plates in SFM containing 20 ng ml^−1^ EGF [30]. After a 5-day-incubation period, the numbers of primary neurospheres were counted under the microscope. For self-renewal assays, neurospheres were reseeded to obtain secondary neurospheres.

SVZ cell culture growth was measured on SVZ neurospheres maintained for 5 days in SFM (control) or SFM supplemented with either 1 μg ml^−1^ warfarin or 10 μg ml^−1^ vitamin K1 or the various SVZ cell culture conditioned media prepared as described above or 20 μg ml^−1^ of antibodies neutralizing protein S (Dako, Glostrup, Denmark, http://www.dako.com) or 20 μg ml^−1^ of an irrelevant antibody (rabbit anti-glial fibrillary acidic protein [GFAP], DakoCytomation). At the end of the assay, viable cells were counted using the trypan blue exclusion assay.

### Detection of SVZ Cells Apoptosis by TUNEL Assays

SVZ neurosphere cultures were maintained for 24 hours in SFM supplemented or not with 1 μg ml^−1^ warfarin. Apoptosis was evaluated by the terminal deoxynucleotidyl transferase dUTP nick end labeling (TUNEL) assay as described [13].

### SVZ Cell Proliferation Assays

SVZ cell cultures were treated for 24 hours with the agents under study. SVZ cell proliferation was evaluated by bromodeoxyuridine (BrdU) incorporation in DNA. BrdU of 10 μM (Sigma-Aldrich, St. Quentin Fallavier, France, http://www.sigmaaldrich.com) was added to the medium for the last 4 hours of the assay. The cells were then processed for BrdU immunostaining [13] using a monoclonal rat anti-BrdU antibody (Harlan Sera-Lab, Leicestershire, United Kingdom, http://www.harlanseralab.co.uk).

### RT-PCR Analysis

Total RNA was prepared from SVZ cell cultures using the RNeasy kit (Qiagen, Hilden, Germany, http://www.qiagen. com). RNA of 2 μg was reverse transcribed at 37°C for 1 hour 30 minutes in a total volume of 25 μl containing 6 units of using Moloney murine leukemia virus-reverse transcriptase (Promega), 0.5 μM DNTPs, and 10 ng of random primers. Polymerase chain reaction (PCR) reactions were performed using Go*Taq* polymerase and the primers described in Supporting Information Table 1. RNA extracted from rats testis, liver, or choroïd plexus was used as positive controls for, respectively, the expression of TAM receptors, coagulation factor, and gas6 [26, 31]. Negative controls were performed in the absence of reverse transcription.

### Western Blotting and Immunoprecipitation Analysis

SVZ cell cultures, SVZ conditioned media, or barium citrate precipitates were homogenized in the Laemmli sample buffer. Proteins were separated on 10% (vol/vol) SDS-polyacrylamide gels and transferred to polyvinylidene difluoride membranes (Immobilon-P, Millipore) and immunoblotted with polyclonal rabbit anti-Gas6 antibody (1/2,000; generous gift from Dr Michael Hall), polyclonal rabbit anti-Protein S (1 μg ml^−1^, Dako, Glostrup, Denmark, http://www.dako.com), monoclonal mouse anti-γ-carboxyglutamate acid residues (1 μg ml^−1^; American diagnostica), polyclonal goat anti-Tyro3 (0.4 μg ml^−1^; Santa Cruz Biotechnologie, http://www.scbt.com), polyclonal goat anti-Axl (0.4 μg ml^−1^; Santa Cruz Biotechnologie, http://www.scbt.com), polyclonal rabbit anti-PhosphoAxl (1 μg ml^−1^; Santa Cruz Biotechnologie, http://www.scbt.com), or monoclonal mouse anti-Phosphotyrosine (0.2 μg ml^−1^; Sigma). For analysis of TAM receptors activation, 5.10^6^ SVZ cells were incubated with 10 μg ml^−1^ of either Gas6 and/or Protein S for 8 minutes at 37°C. Both Tyro3 or Mer were immunoprecipitated using 1 μg of anti-Tyro3 or anti-Mer antibodies per 100 μg of total proteins followed by addition of 30 μl of protein G sepharose beads. Axl receptor phosphorylation was directly analyzed by Western blotting using antiphospho Axl antibodies. The bands on the blots were quantified and values were presented as means ± SEM. The statistical evaluation of the data was performed with analysis of variance (ANOVA).

The predicted molecular mass of murine Gas6 is 62 kDa. The observed molecular mass of commercially available recombinant murine Gas6 produced by murine cells is 72 kDa (R&D Systems, cat number 986-GS) owing to its level of post-translational modifications. For our Western blotting analysis of Gas6 secretion by cultured SVZ cells, we used as a positive control, recombinant murine Gas6 produced by human HEK293 cells, as described above. The observed and reported molecular mass of recombinant murine Gas6 produced by human HEK293 cells is 85 kDa [31], owing most likely to a higher level of murine Gas6 post-translational modifications by human HEK293 cells [31].

### Intracerebroventricular Injections and Brain Tissue Processing

Four-month-old mice were anesthetized with 300 mg kg^−1^ of chloral hydrate. In a first set of experiments, 1 μl of either 100 mg ml^−1^ warfarin (Sigma) diluted in a 9-mg ml^−1^ NaCl solution or 9 mg ml^−1^ NaCl solution was injected as a single dose in the left cerebral ventricle using a 5-μl Hamilton syringe at the following coordinates (anterior relative to Bregma, lateral, depth below the dura): 0.74, 0.6, and 2.18 mm. In a second set of experiments, 1 μl of a polyclonal rabbit anti-protein S antibody (40 μg ml^−1^, DakoCytomation) or of an irrelevant rabbit anti-GFAP antibody (40 μg ml^−1^, DakoCytomation) was injected in the left cerebral ventricle. BrdU (50 mg kg^−1^ of body weight) was administered intraperitoneally 4 hours before mouse sacrifice. Approximately 72 hours postintracerebroventricular injections, mice were anesthetized and transcardially perfused with a 9% (vol/vol) NaCl solution followed by 4% (vol/vol) paraformaldehyde. Brains were removed and postfixed in 4% paraformaldehyde at 4°C overnight. Frontal brain sections, 40 μm thick, were cut using a Leica vibratome and processed for BrdU immunohistodetection as described [13].

Coimmunostaining was performed by incubating the brain sections in 5 μg ml^−1^ monoclonal rat anti-BrdU antibody and either 10 μg ml^−1^ monoclonal chicken anti-GFAP (Abcam, Cambridge, U.K., http://www.abcam.com) or 2 μg ml^−1^ polyclonal goat anti-doublecortin (Santa Cruz) antibodies. Brain sections were then incubated with biotinylated anti-rat antibody followed by avidine fluorescein and goat anti-chicken Alexa fluor 555 or donkey anti-goat Alexa fluor 555 as appropriate. Preparations were analyzed with a spectral confocal FV-1000 station installed on an inverted microscope IX-81 (Olympus) and each labeled cell was examined along the *z*-axis to ensure proper identification of double labeled cells.

### Immunostaining

Immunostaining of Gas6, protein S, Tyro 3, Mer, or Axl was performed by incubating the brain sections in polyclonal rabbit anti-Gas6 (1/100; generous gift from Dr Michael Hall) or polyclonal rabbit anti-Protein S (40 μg ml^−1^, DakoCytomation) or polyclonal goat anti-Tyro3 (2 μg ml^−1^, Santa Cruz), polyclonal goat anti-Mer (2 μg ml^−1^, Santa Cruz), or polyclonal goat anti-Axl (2 μg ml^−1^, Santa Cruz). Coimmunostainings were performed with either monoclonal rat anti-CD31 (2.5 μg ml^−1^; BD Biosciences) or polyclonal goat anti-doublecortin (0.8 μg ml^−1^, Abcam) or monoclonal chicken anti-GFAP (10 μg ml^−1^, Abcam), polyclonal rabbit anti-GFAP (3 μg ml^−1^, DakoCytomation) or monoclonal mouse antinestin (10 μg ml^−1^, Chemicon) or monoclonal mouse anti βIII tubulin (30 μg ml^−1^, Sigma) antibody. Brain sections were then incubated with the following antibodies as appropriate: goat anti-rabbit Alexa fluor 488 or goat anti-mouse Alexa fluor 555 or goat anti-chicken Alexa fluor 555 or donkey anti-rabbit Alexa fluor 488 or donkey anti-goat Alexa fluor 555 or donkey anti-mouse Alexa fluor 647 or goat anti-rat Alexa fluor 488 or biotinylated goat anti rabbit followed by avidine TRITC. DAPI was used to label nuclei.

### Labeling of Slowly Dividing SVZ Stem-Like Cells in Wild-Type and Gas6^−/−^ Mice

Two-month-old wild type and Gas6^−/−^ mice were subjected to daily intraperitoneal injections of 50 mg kg^−1^ body weight of BrdU for 5 days. After 28 days of first injection, brains were fixed and BrdU-positive cells were quantified in the SVZ as above.

### Analysis of Olfactory Bulb Neurogenesis in Wild-Type and Gas6^−/−^ Mice

Three-month-old wild type and Gas6^−/−^ mice were subjected to three intraperitoneal injections of 50 mg kg^−1^ BrdU with a 2-hour interval between each injection during 1 day. Three weeks after injection, brains were fixed and 14 μm thick olfactory bulb sections were cut and processed for BrdU immunocytochemistry. Data were collected with the help of mapping software (Mercator Pro, Explora Nova), coupled to a Zeiss microscope. All BrdU-positive cells were counted in the granule cell layer of four sections (280 μm between each section) of the right olfactory bulb in both experimental groups. Density of BrdU-positive cells (number of labeled profiles/μm^2^) was calculated for each layer.

The phenotype of BrdU-positive cells in the granule cell layer of the olfactory bulb was determined by immunostaining of BrdU along with a mouse anti-NeuN (1:500; Chemicon) antibody and analyzing doublelabeling by pseudoconfocal scanning microscopy (apotome, Zeiss). Each labeled cell was examined along the *z*-axis to ensure proper identification of double labeled cells.

### ELISA of Soluble Axl

Soluble Axl was detected using enzyme-linked immunosorbent assay (ELISA) on SVZ cells conditioned media according to manufacturer's instructions (R&D systems).

### Data Analysis

Cell number counts were performed by using the Neurolucida system for image analysis (MicrobrightField Inc) coupled to a BX60 Olympus microscope. Percentages of TUNEL-, BrdU-, and immunoreactive cells were determined from cell counts in 10 fields of view in each coverslip with a 40× objective, by an observer blind to the assays (more than 400 cells were counted per coverslip).

For intracerebroventricular injection experiments and for the quantification of slowly dividing SVZ stem-like cells in wild-type and Gas6^−/−^, numbers of BrdU-positive cells were scored in the SVZ and, when specified in the area adjacent to SVZ (region 2, Fig. 2), by an observer blind to the assays on five regularly spaced brain sections sampled at similar anatomical levels between 1.18 and 0.14 anterior to Bregma and are expressed as means ± SEM per section. Statistical significance of differences was examined by one-way ANOVA followed by the post hoc Bonferroni test for multiple comparisons or by non-parametric Mann and Whitney test for comparison by pairs (Statview 5.00 software). Statistical significance level was set for *p* < .05.

## RESULTS

### Administration of the Vitamin K Antagonist Warfarin Promotes SVZ Cell Proliferation

**Figure 1:**
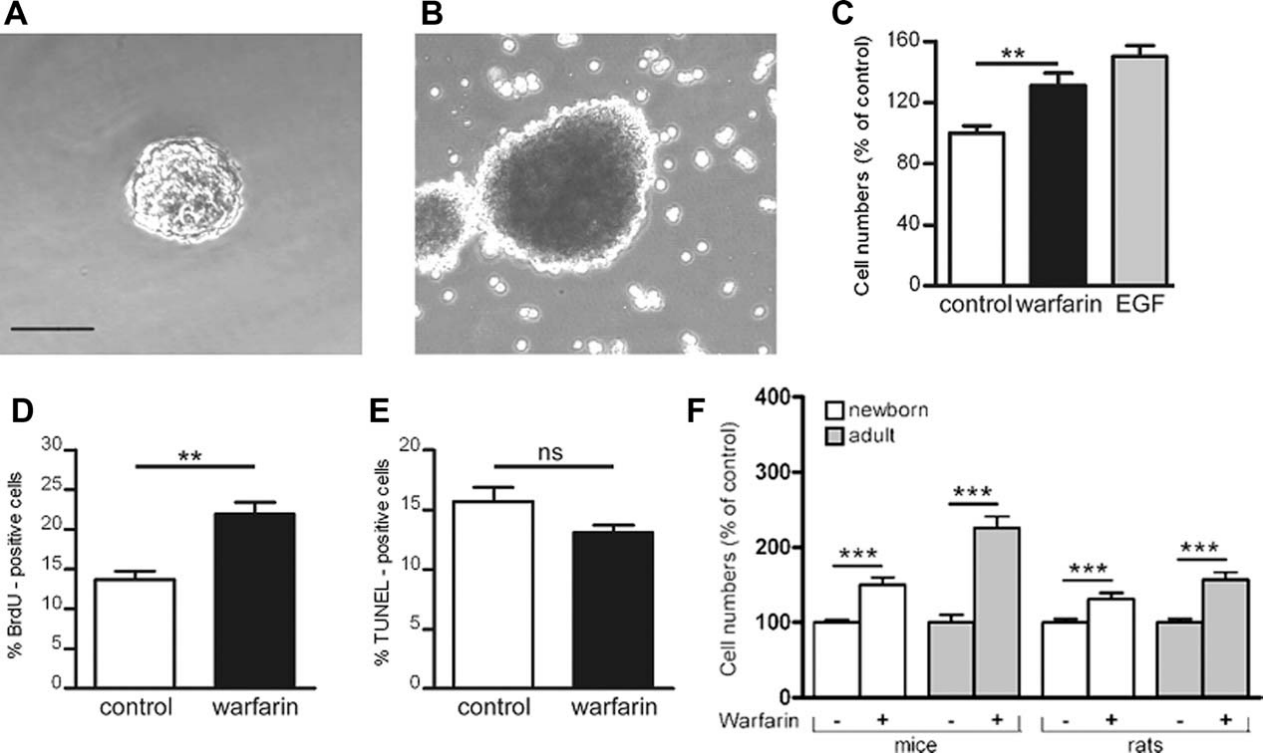
Regulation of subventricular zone (SVZ) cell culture growth, proliferation, and apoptosis by warfarin ((S(−)-3-acetonylbenzyl)-4-hydroxycoumarin)). Representative photographs of SVZ neurospheres maintained for 5 days either in serum-free medium (SFM) **(A)** or in SFM supplemented with 1 μg ml^−1^ warfarin **(B)**. Scale bar = 200 μm. **(C):** Growth of SVZ cell cultures maintained for 5 days in either SFM (control) or in SFM supplemented with either 1 μg ml^−1^ warfarin or with 20 ng ml^−1^ epidermal growth factor. Percentages of bromodeoxyuridine- **(D)** or terminal deoxynucleotidyl transferase dUTP nick end labeling-stained **(E)** nuclei in SVZ cell cultures maintained for 24 hours in SFM (control) or SFM supplemented with 1 μg ml^−1^ warfarin. **(F):** Growth of SVZ cell cultures maintained for 5 days in either SFM (control) or SFM supplemented with 1 μg ml^−1^ warfarin. SVZ cells were derived from newborn or adult rats or mice. Data were obtained from at least three independent experiments each in quadruplicates and are expressed in **(C)** and **(F)** as percentages of total viable cell numbers in SFM ± SEM, in **(D)** and **(E)** as percentages ± SEM. We used Mann-Whitney test for data statistical analysis. ***, *p* < .0001; **, *p* < .01; ns, *p* = .0570. Abbreviations: BrdU, bromodeoxyuridine; EGF, epidermal growth factor; TUNEL, terminal deoxynucleotidyl transferase dUTP nick end labeling.

We maintained SVZ cells obtained from 1 to 3 days old Wistar rats for 5 days in a SFM (control) or in a SFM supplemented either with 1 μg ml^−1^ warfarin or with 20 ng ml^−1^ EGF, a known potent SVZ cell mitogen [32, 33]. Exposure to warfarin led to a significant increase in SVZ cell number with an extent similar to that induced by EGF (Fig. 1A–1C). Warfarin induced SVZ cell culture growth over a concentration range of 0.1–10 μg ml^−1^ with a substantial response at 1 μg ml^−1^ (Supporting Information Fig. 1 online), a concentration used in all subsequent experiments. BrdU cell proliferation and TUNEL cell apoptosis assays indicated that warfarin-induced SVZ cell number increase was due to a stimulatory effect of warfarin on cell proliferation rather than on cell survival (Fig. 1D, 1E). As in rodents, the effects of some mitogens depend on the species or on the age of the animals from which SVZ cell cultures are derived [34, 35], we tested and established that warfarin administration significantly promotes the growth of SVZ cell cultures derived from both mice and rats both newborn or adult (Fig. 1F).

**Figure 2:**
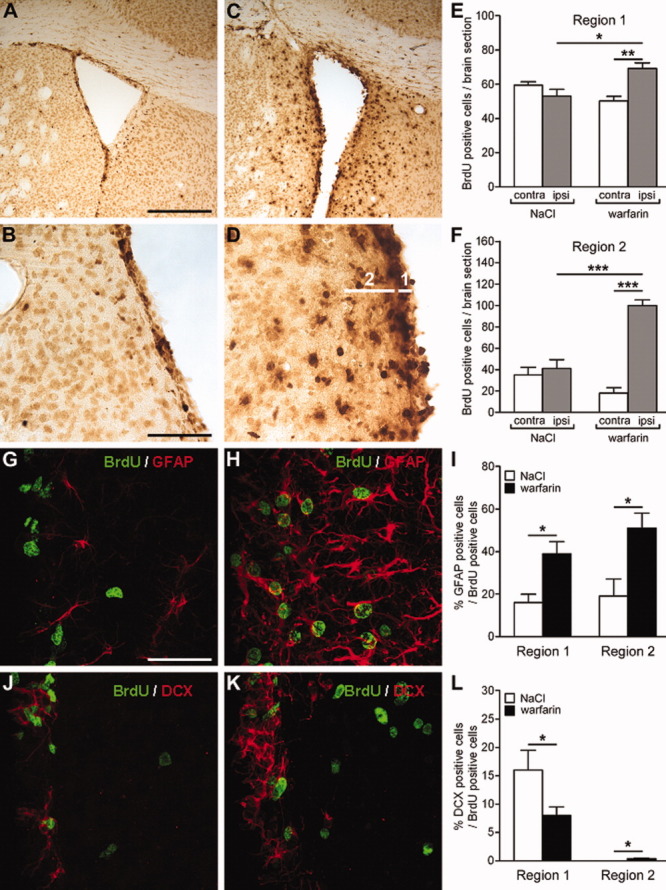
Intracerebroventricular injection of warfarin ((S(−)-3-acetonylbenzyl)-4-hydroxycoumarin)) stimulates cell proliferation in vivo in the regions surrounding the lateral ventricles. Immunostaining of bromodeoxyuridine (BrdU) (in dark, **A–D)** in brain sections following a single injection of NaCl (1 μl, 9 mg ml^−1^) **(A, B)** or warfarin (1 μl, 100 mg ml^−1^) **(C, D**) in the lateral ventricle of mice. Mice were subjected to intraperitoneal injections of BrdU (50 mg kg^−1^) 68 hours later and sacrificed 72 hours postintracerebroventricular injections. Scale bars = 1 mm **(A, C)**; 200 μm **(B, D)**. In **(E)** and **(F)**: BrdU-positive cells were quantified within and in the region (region 2) adjacent to the subventricular zone on sections sampled between 1.18 and 0.14 mm anterior to Bregma, on hemispheres contralateral (contra) or ipsilateral (ipsi) to warfarin or NaCl injection. Means ± SEM numbers of BrdU-positive cells per brain section obtained in regions 1 and 2 from six 4-month-old mice per experimental condition were analyzed with one-way analysis of variance (ANOVA) followed by the post hoc Bonferroni test. *, *p* < .05; **, *p* < .01; ***, *p* < .0001. Double immunostaining for BrdU (green) and glial fibrillary acidic protein (GFAP) (red, **G** and **H)** or doublecortin (red, **J** and **K)** in ipsilateral brain hemispheres of mice injected with NaCl **(G, J)** or warfarin **(H, K)**. Scale bar = 40 μm. Each labeled cell was examined along the *z*-axis to ensure proper identification of double labeled cells. Percentages of GFAP-**(I)** or Dcx-over BrdU **(L)**-positive cells in ipsilateral brain hemispheres of mice injected with NaCl (white bars) or with warfarin (dark bars). Means ± SEM obtained from six 4 months old mice per experimental condition were analyzed with one-way ANOVA followed by the post hoc Bonferroni test. *, *p* < .05; **, *p* < .01; ***, *p* < .0001. Abbreviations: BrdU, bromodeoxyuridine; DCX, doublecortin; GFAP, glial fibrillary acidic protein; ipsi, ipsilateral.

Next, we subjected mice to a direct single intracerebroventricular injection of warfarin (1 μl, 100 mg ml^−1^ diluted in NaCl 9 mg ml^−1^) or NaCl (1 μl, 9 mg ml^−1^) in the lateral ventricle and to a single BrdU (50 mg kg^−1^) intraperitoneal injection 68 hours later. We sacrificed mice 72 hours postintracerebroventricular injections and analyzed brain sections. When compared with control saline-injected, warfarin-injected brains displayed a massive increase in BrdU incorporating cells in the regions surrounding the lateral ventricles (Fig. 2A–2F and Supporting Information Fig. 2 online). Double-immunostaining experiments of BrdU along with doublecortin (Dcx) and GFAP, which are specific cell markers for migrating neuroblasts and glial or stem cells, respectively [36, 37], indicated that warfarin specifically promoted the proliferation of GFAP expressing cells and reduced the proportion of Dcx-positive cells among the dividing cell population (Fig. 2), implying that the inhibition of functional VKDP production suppresses an endogenous inhibitory mechanism for SVZ cell proliferation and promotes SVZ cells preferentially toward a glial or stem-like fate.

### Vitamin K and SVZ Endogenous VKDPs Counterbalance Warfarin-Induced SVZ Cell Proliferation

**Figure 3:**
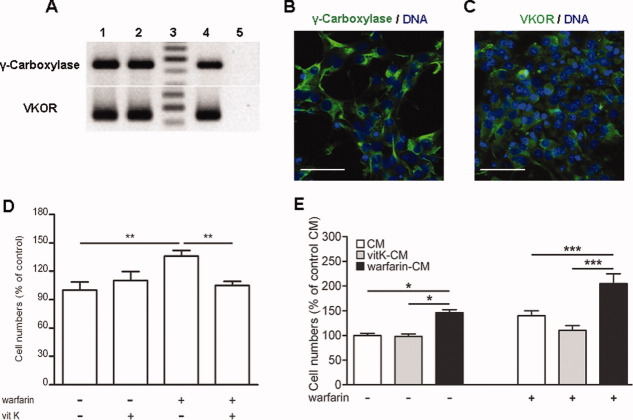
Subventricular zone (SVZ) cells express the gamma carboxylase and VKOR enzymes and produce active vitamin K-dependent proteins that reverse warfarin ((S(−)-3-acetonylbenzyl)-4-hydroxycoumarin)) effects. **(A):** Real time-polymerase chain reaction (RT-PCR) detection of the γ-carboxylase and VKOR transcript in SVZ cells. RT-PCR analysis of RNA obtained from reverse transcriptase two different (lanes 1 and 2) SVZ cell cultures. Lane 3 corresponds to DNA molecular weight markers. Lane four depicts RT-PCR products obtained using RNA from tissues (liver) known to express both transcripts (positive control). Lane 5 represents RT-PCR performed in the absence of reverse transcription (negative control). Micrographs representing immunostaining of SVZ cell cultures with either anti-γ-carboxylase **(B**, in green) or anti-VKOR **(C**, in green) antibody. Cell nuclei were labeled with TOPRO-3 (blue). Scale bar = 20 μm. **(D)** Growth of SVZ cell cultures maintained for 5 days in either serum-free medium (SFM; control) or in the presence of 1 μg ml^−1^ warfarin or 10 μg ml^−1^ vitamin K1 (vit K) or a combination of both. **(E)** Growth of SVZ cell cultures maintained for 5 days in SFM (control) or in SFM supplemented with either 1 μg ml^−1^ warfarin or conditioned media derived from SVZ cell cultures that had been treated for 5 days in either SFM (CM) or 10 μg ml^−1^ vitamin K1 (vitK-CM) or 1 μg ml^−1^ warfarin (warfarin-CM) or a combination of the above. Data were obtained from at least three independent experiments each in quadruplicates and are expressed as percentages of total viable cell numbers in SFM ± SEM in **(D)** or of total viable cells in CM in **(E)**. We performed statistical analysis by one-way analysis of variance followed by the post hoc Bonferroni test for multiple comparisons. *, *p* < .05; **, *p* < .01; ***, *p* < .0001. Abbreviations: CM, conditioned media; VKOR, vitamin K epoxide reductase.

Real time-PCR (reverse transcriptase (RT)-PCR; Fig. 3A) together with immunostaining analysis (Fig. 3B, 3C) shows that SVZ cells express the gamma carboxylase and the vitamin K epoxide reductase enzyme system and hence could produce functional VKDPs. Warfarin mitogenic effect on SVZ cell cultures is antagonized by the addition of vitamin K, confirming that it is specifically due to the inhibition of endogenous functional VKDPs production (Fig. 3D). To investigate if the suppression by warfarin of endogenous VKDP production is responsible of warfarin growth stimulatory effect on SVZ cell cultures, we prepared conditioned media from SVZ cell cultures either enriched in- or depleted from- secreted VKDPs by maintaining SVZ cell cultures for 5 days in SFM or in SFM containing either 10 μg ml^−1^ vitamin K1 or 1 μg ml^−1^ warfarin. All conditioned media were ultrafiltrated by centrifugation on an YM-3 Centricon filter device to remove residual vitamin K1 or warfarin and we confirmed their effective removal by HPLC analysis (Supporting Information Fig. 3). The upper fractions (MW >3,000 Da) were recovered, normalized to cell numbers from which they were derived by dilution in SFM and used for SVZ cell culture growth assays. We used the activity of conditioned media obtained in SFM as a baseline for the assay and expressed the data as percentage of this activity. Conditioned media from vitamin K1-treated cells significantly reduced SVZ cell proliferation induced by warfarin while conditioned media from warfarin-treated cells stimulated cell proliferation by its own and accentuated the effects of warfarin (Fig. 3E). Therefore, the secretion of functional VKDPs leads to an inhibition of SVZ cell proliferation and its suppression could account for warfarin growth stimulatory effects on SVZ cell cultures (Fig. 1).

### SVZ Cells Produce Gas6 and Protein S and Express Tyro3 and Axl Receptors

**Figure 4:**
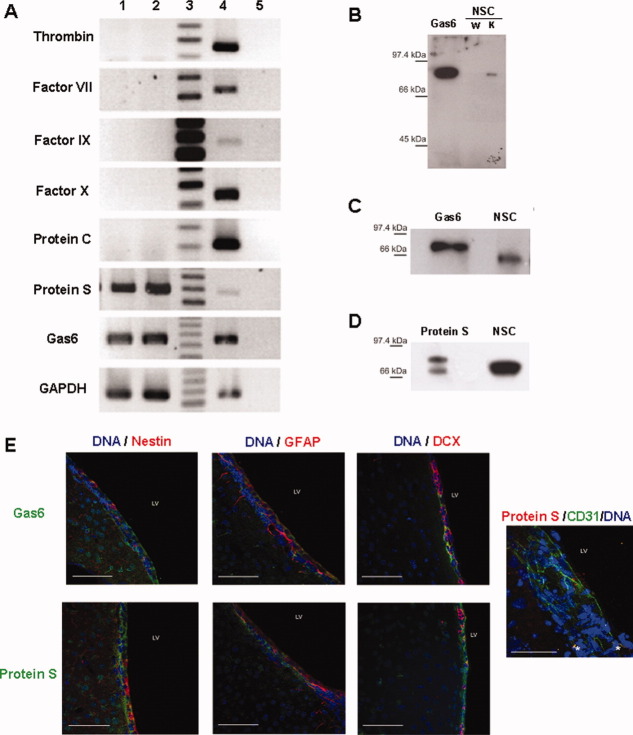
Subventricular zone (SVZ) cells produce Gas6 and protein S in vitro and in vivo. **(A)** Real time-polymerase chain reaction (RT-PCR) detection of secreted vitamin K-dependent proteins (VKDPs) in SVZ cells cultures. RT-PCR reverse transcriptase analysis of RNA obtained from two different (lanes 1 and 2) SVZ cell cultures using oligonucleotides specific for the indicated VKDP c-DNA or GAPDH used as a control. Lane 3 corresponds to DNA molecular weight markers. Lane 4 depicts RT-PCR products obtained using RNA from tissues known to express the indicated VKDP transcripts (positive control) and which are liver for thrombin, factor VII, IX, and X, protein C, protein S, and choroid plexus for Gas6. Lane 5 represents RT-PCR performed in the absence of reverse transcription (negative control). Western blotting analysis of conditioned media obtained from warfarin ((S(−)-3-acetonylbenzyl)-4-hydroxycoumarin)) (W) or vitamin K1 (K) treated SVZ cells **(B-D)** using either an anti-γ-carboxyglutamate residues antibody **(B)** or an anti-Gas6 antibody **(C)** or an anti-protein S antibody **(D)**. Purified human protein S or murine Gas6 produced by human HEK293 cells, as described in the material and method section were used as positive controls. **(E)** Mice brain sections from the SVZ were doubleimmunostained using anti-protein S (green) or anti-Gas6 (green) antibody together with anti-nestin (red), anti-glial fibrillary acidic protein (red) or anti-doublecortin antibody. Cell nuclei were stained with DAPI (blue). Scale bar = 60 μm. Brain sections were also stained with anti-protein S (red) and anti-CD31 (green) antibody. Asterisks show protein S localization in blood vessel. Scale bar = 40 μm. Abbreviations: DCX, doublecortin; GAPDH, glyceraldehade-3-phosphate dehydrogenase; GFAP, glial fibrillary acidic protein; LV, lateral ventricle; NSC, neural stem cell.

With an aim to identify the endogenous VKDP secreted by SVZ cells, we first determined by PCR which of the eight known secreted VKDPs [16] may be expressed at transcript level by SVZ cell cultures. Only transcripts corresponding to the anticoagulant factors, protein S and its structural homolog Gas6, are expressed by SVZ cells (Fig. 4A). We then analyzed SVZ cell conditioned media obtained from warfarin- and vitamin K1-treated cells by Western blotting using an anti-γ-carboxyglutamate residue antibody, which specifically recognizes proteins with the γ-carboxylation modification [38]. We detected in conditioned media from vitamin K1-treated but not from warfarin-treated SVZ cell cultures a unique VKDP band at 80 kDa (Fig. 4B). Protein S and its structural homolog Gas6 are secreted VKDPs with molecular weights in the 69–80 kDa range and could both correspond to the γ-carboxylated protein detected in Figure 4B. Western blotting analysis using either anti-protein S or anti-Gas6 antibodies, revealed the presence of both Gas6 (Fig. 4C) and protein S (Fig. 4D) in SVZ cell culture conditioned media. Immunostaining analysis confirmed the expression of both Gas6 and protein S by cultured SVZ cells (Supporting Information Fig. 4). Furthermore, analysis of mice brain sections confirmed the presence of both Gas6 and protein S in the SVZ (Fig. 4E, Supporting Information Fig. 5). Protein S or Gas6 were mostly detected within GFAP- or nestin-expressing cells but not in neuroblasts (Dcx-positive cells). As expected, protein S was also found in endothelial cells (CD31-positive) (Fig. 4E).

**Figure 5:**
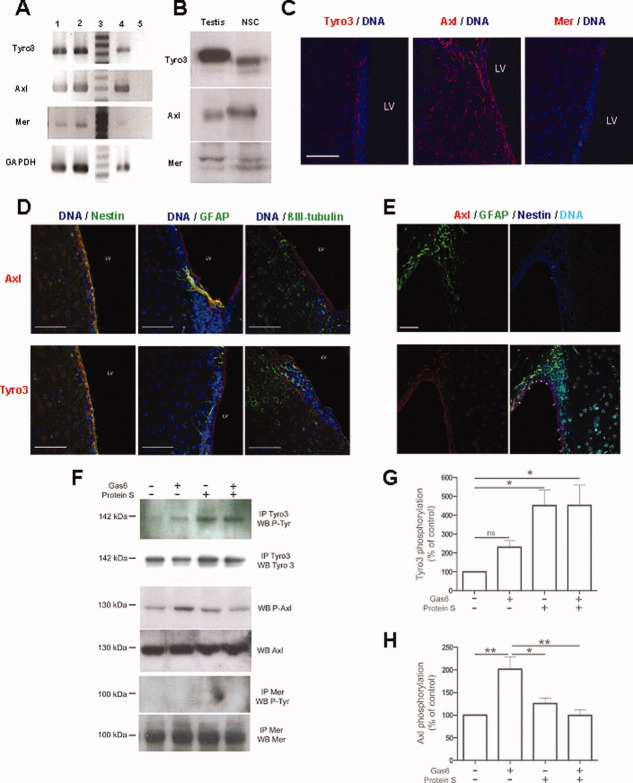
Subventricular zone (SVZ) cells express Tyro3, Axl, and Mer (TAM) receptors that are activated by Gas6 or protein S. **(A)** Real time-polymerase chain reaction (RT-PCR) analysis of RNA obtained from two different (lanes 1 and 2) SVZ cell cultures using oligonucleotides reverse transcriptase specific for TAM or GAPDH used as a control. Lane 3 corresponds to DNA molecular weight markers. Lane 4 corresponds to RT-PCR products obtained using RNA from a tissue (testis) known to express TAM receptors (positive control) and lane 5 to RT-PCR performed in the absence of reverse transcription (negative control). **(B)** Western blotting analysis of 50–100 μg of protein of SVZ cell lysates (neural stem cell) using either anti-Tyro3, anti-Axl, or anti-Mer antibodies. Protein lysates (50–100 μg) from testis were used as positive control. **(C):** Immunostaining (red) of Axl, Tyro3, or Mer on SVZ mice brain sections. Scale bar = 60 μm. **(D, E):** Coimmunostaining of Axl (red) or Tyro3 (red) along with nestin or glial fibrillary acidic protein (GFAP) or βIII tubulin (green, **D)** or GFAP (green, **E)** and nestin (blue, **E**). Cell nuclei were stained with DAPI (blue **D** or cyan in **E**). Scale bar = 60 μm. **(F–H):** Western blotting analysis of cell lysates obtained from SVZ cell cultures maintained in serum-free medium (SFM) or in SFM supplemented with 10 μg ml^−1^ of Gas6, Protein S or both for 8 minutes. Cell lysates of 80 μg were either immunoprecipitated with anti-Tyro3 or anti-Mer antibody and immunoprecipitates analyzed by Western blotting (WB) with antiphosphotyrosine (P-Tyr), anti-Tyro3, and anti-Mer or were directly analyzed by Western blotting using either an anti-phospho-Axl antibody (P-Axl) or an anti-Axl antibody. The bands on the blots were quantified by densitometry and values were presented as means ± SEM. The statistical evaluation of the data was performed with ANOVA. * *p* < .05, ** *p* < .01. Abbreviations: GAPDH, glyceraldehade-3-phosphate dehydrogenase; GFAP, glial fibrillary acidic protein.

Gas6 and protein S exert their effects by interacting with the tyrosine kinase receptors TAM for which they are the only known ligands [20]. We determined by PCR analysis (Fig. 5A), by Western blotting (Fig. 5B) and immunostaining (Supporting Information Fig. 6) that SVZ cell cultures express TAM. Analysis of mice brain sections revealed that in vivo, Axl and Tyro3 are expressed within the SVZ (Fig. 5C). Mer was only detected at a very low level that precluded any further analysis. Axl is expressed by immature cells (nestin-positive) and by some cells expressing both GFAP and nestin, which is a feature of stem cells (Fig. 5D, 5E). Tyro-3 is mostly expressed by immature cells (nestin-positive cells), neuroblasts (βIII tubuline-positive cells), and by few GFAP-positive cells (Fig. 5D).

**Figure 6:**
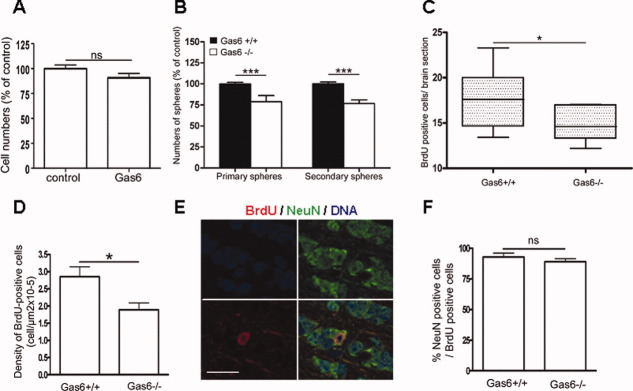
Gas6 knock out reduces the numbers of subventricular zone (SVZ) cells with stem-like cell properties. **(A):** Growth of SVZ cell cultures maintained for 5 days in serum-free medium (SFM) (control) or in SFM supplemented with 1 μg ml^−1^ Gas6. **(B):** Numbers of primary neurospheres, obtained after incubating SVZ cells derived from wild type (black box) or Gas6^−/−^ (white box) for 5 days in SFM supplemented with 20 ng ml^−1^ epidermal growth factor (EGF) are depicted. Neurospheres obtained in each of the conditions were harvested, dissociated as single cells, replated in SFM supplemented with 20 ng ml^−1^ EGF for 5 additional days and the numbers of secondary neurospheres are also depicted. **(C):** Numbers of slowly dividing SVZ stem-like cells in wild type or Gas6^−/−^ mice were obtained by subjecting mice to daily intraperitoneal injections of 50 mg kg^−1^ bromodeoxyuridine (BrdU) for 5 days and analyzing 24 days later BrdU-positive cells in the SVZ. **(D):** Numbers of newly generated cells that joined the olfactory bulb in wild-type or Gas6^−/−^ mice were obtained by subjecting mice to three intraperitoneal injections of 50 mg kg^−1^ BrdU with a 2-hour interval between each injection during 1 day and analyzing 24 days later, the numbers of BrdU-positive cells within the olfactory bulb. **(E):** Double immunostaining of BrdU-positive cells with the neuronal marker NeuN in the olfactory bulb, scale bar = 8 μm. **(F):** Percentages of NeuN (neuronal marker)-positive cells among the BrdU-positive cells in the olfactory bulb in wild-type and Gas6^−/−^ mice. Data were obtained from at least three experiments **(A, B)** or six mice **(C, D, F)** and are depicted as means of percentages of total cell numbers in **(A)** or numbers of immunoreactive cells per brain section in **(C)** or density of BrdU-positive cells in **(D)** or percentages of NeuN-positive cells among BrdU-positive cells in **(F)**. Mann-Whitney test was used for data statistical analysis; ns, *p* > .05 for **(A)** and **(F)**; *, *p* < .05 for **(C, D)**. Abbreviation: BrdU, bromodeoxyuridine.

As both the ligands (protein S and Gas6) and the receptors (TAM) are expressed by SVZ cells, we analyzed the activation pattern of TAM receptors by either Gas6, protein S, or their combined addition. Tyro3 receptor is significantly activated by protein S but not by Gas6 and the combined addition of Gas6 and protein S does not alter the activation level of Tyro3 by protein S (Fig. 5F–5H). Axl is significantly activated by Gas6 but not by protein S; however, the coaddition of Gas6 and protein S suppresses Axl activation (Fig. 5F–5H). We failed to detect Mer receptor activation by either Gas6, protein S, or their combined addition. As a soluble form of Axl was described [39], we performed ELISA that can detect as little as 1 ng ml^−1^ of soluble Axl but which did not reveal the presence of soluble Axl in either SVZ cells conditioned media both unconcentrated and fivefold-concentrated. Together these data suggest that Gas6 activates Axl, and that protein S exerts its cellular effects on SVZ cells by activating Tyro3 pathway and inhibiting Gas6-induced Axl activation.

### Loss of Function Experiments Reveals a Dual Role of Gas6 and Protein S in Regulating SVZ Cell Proliferation

The fact that warfarin, by suppressing the secretion of functional endogenous VKDPs, stimulates both in vitro and in vivo SVZ cell proliferation (Fig. 1 and 2), implies that under basal conditions secreted VKDP constitutively inhibit SVZ cell proliferation. As protein S and its structural homolog Gas6 are the two only VKDPs and secreted by SVZ cells, we next investigated which of the two endogenous VKDP, Gas6 or protein S, could be responsible for the inhibition of SVZ cell proliferation.

Exogenously added Gas6 for concentrations up to 10 μg ml^−1^ did not inhibit SVZ cell proliferation (Fig. 6A). As SVZ cells produce Gas6 but do not seem to respond to it, we studied SVZ cells behavior in Gas6^−/−^ mice [27]. Numbers of SVZ primary and secondary neurospheres were significantly lower in Gas6^−/−^ mice when compared with wild-type mice (Fig. 6B), suggesting that within the SVZ, the suppression of Gas6 expression reduces more specifically stem cell proliferation or/and stem cells pools. Therefore, we traced slowly dividing SVZ stem cells in vivo in wild type and Gas6^−/−^ mice, by subjecting mice to daily intraperitoneal injection of 50 mg kg^−1^ BrdU for 5 days and analyzing 24 days later BrdU-positive cells within the SVZ. Numbers of long-term label retaining cells in the SVZ were significantly lower in Gas6^−/−^ mice (Fig. 6C). Under physiological conditions, SVZ stem cells provide a continuous supply of new neurons in the olfactory bulb [2]. If endogenous Gas6 plays an important functional role for SVZ stem cells pool maintenance or proliferation, we should expect less new neurons in the olfactory bulb of Gas6^−/−^ mice. We traced newly generated olfactory bulb neurons in wild type or Gas6^−/−^ mice by subjecting mice to three intraperitoneal injections of 50 mg kg^−1^ BrdU with a 2-hour interval between each injection during 1 day, and analyzed 24 days later, within the olfactory bulb, BrdU-positive cells and phenotyped these cells with the specific neuronal marker NeuN [40, 41]. We observed that the ratio of neurons (NeuN positive) among BrdU-positive cells did not vary between wild type and Gas6^−/−^ mice (Fig. 6F). However, when compared with wild type, in Gas6^−/−^ mice, the numbers of BrdU-positive cells in the olfactory bulb are significantly lower, implying that Gas6 deletion results in significant decrease in the olfactory bulb neurogenesis (Fig. 6D–6F). Together, our data suggest that Gas6 promotes stem cell proliferation and/or pools maintenance and rule out Gas6 as the endogenous VKDP responsible for inhibiting SVZ cell proliferation.

**Figure 7:**
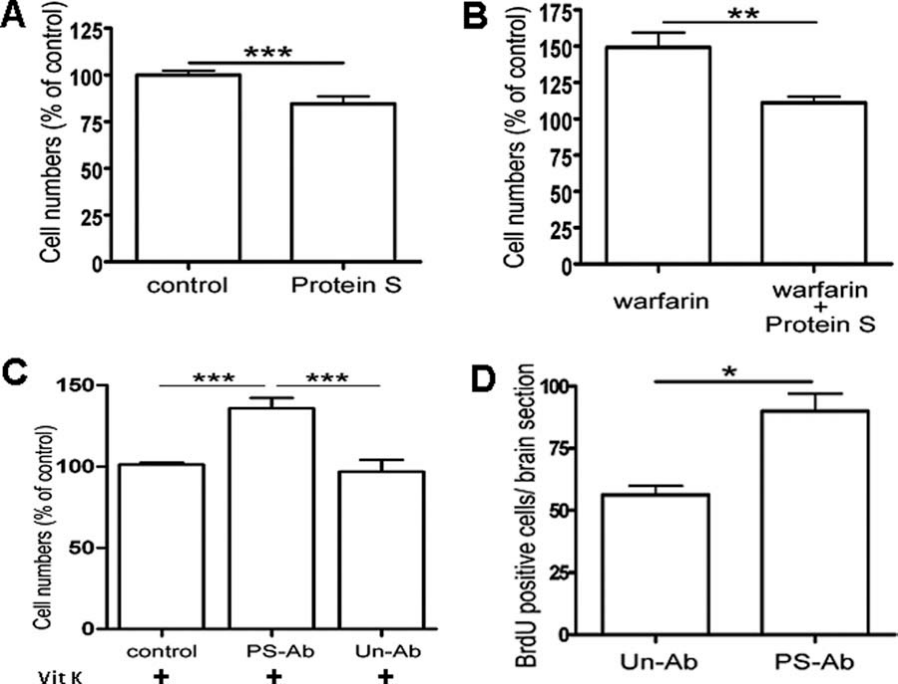
Protein S inhibits subventricular zone (SVZ) cell proliferation and reverses warfarin ((S(−)-3-acetonylbenzyl)-4-hydroxycoumarin)) effects. **(A):** Growth of SVZ cell cultures maintained for 5 days in serum-free medium (SFM) (control) or in SFM supplemented with 1 μg ml^−1^ protein S. **(B):** Growth of SVZ cell cultures maintained for 5 days in SFM containing 1 μg ml^−1^ warfarin in absence or presence of 1 μg ml^−1^ protein S. **(C):** Growth of SVZ cell cultures maintained for 5 days in SFM containing 10 μg ml^−1^ vitamin K in the absence or in the presence of 20 μg ml^−1^ rabbit anti-protein S or an irrelevant rabbit antibody (anti-glial fibrillary acidic protein). **(D):** Numbers of bromodeoxyuridine (BrdU)-positive cells in the SVZ of 6 months old mice that were subjected to intracerebroventricular injection of anti-protein S or irrelevant antibody (UnAb 1 μl of a 40 μg ml^−1^ solution). Mice received an intraperitoneal injection of BrdU (50 mg kg^−1^) 68 hours later and were sacrificed 72 hours postintracerebroventricular injections. Data were derived from four mice injected with irrelevant (Un-Ab) antibody and three with anti-protein S. Data were obtained from at least three independent experiments each in quadruplicates and represent means ± SEM of percentages of total cell numbers in **(A)**, **(B),** or **(C)** or of numbers of immunoreactive cells in the SVZ per brain section in **(D)**. Statistical analysis of the data was performed using the Mann-Whitney test in **(A)**, **(B)**, and **(D)** or the one-way analysis of variance followed by the post hoc Bonferroni test in **C**. *, *p* < .05; **, *p* < .01; ***, *p* < .001. Abbreviations: PS-Ab, anti-protein S antibody; Un-Ab, unrelated antibody.

Exogenously added protein S at 1 μg ml^−1^ inhibits in vitro SVZ cell proliferation (Fig. 7A) and antagonizes warfarin mitogenic effect on SVZ cells (Fig. 7B) mimicking thereby the activity of SVZ cell culture conditioned media obtained in the presence of vitamin K1 (Fig. 3E). Moreover, exposure of SVZ cell culture to 1 μg ml^−1^ protein S during 5 days, significantly increased the proportion of cells in the neuronal lineage (13.9% ± 0.7% cells expressed βIII-tubulin in control condition when compared with 47.8% ± 1.7% in protein S condition; *p* < .01), suggesting that protein S does not only slow down proliferation but also favors neuronal differentiation of cultured SVZ cells. Lethal purpura fulminans develops in the very rare newborns homozygous for protein S gene [42]. Therefore, protein S knockout mice could not be used in our studies to investigate protein S loss of function within the SVZ. However, we used anti-protein S neutralizing antibodies to investigate loss of function linked to endogenous protein S suppression. Neutralization of endogenously produced protein S in vitro by addition of an anti-protein S antibody to cell culture medium for 5 days significantly increased SVZ cell proliferation when compared with cultures treated under similar conditions with an irrelevant antibody (Fig. 7C). With an aim to determine if the inhibitory effect of endogenous protein S on SVZ cell proliferation observed in vitro, could be reproduced in vivo, we subjected mice to a direct single intracerebroventricular injection in the lateral ventricle of either an anti-protein S antibody or an irrelevant antibody (anti-GFAP, 1 μl, 40 μg ml^−1^) followed by a single BrdU (50 mg kg^−1^) intraperitoneal injection 68 hours later. We sacrificed mice 72 hours postintracerebroventricular injections and analyzed brain sections. When compared with controls, brains from anti-protein S antibody injected mice displayed a significant increase in cell proliferation in the SVZ (Fig. 7D). Taken together these data suggest that while endogenous Gas6 promotes SVZ stem cells proliferation and/or pool maintenance, protein S is the endogenous VKDP that is responsible for inhibiting SVZ cell proliferation in vivo.

## DISCUSSION

Using multiple approaches, our study uncovers a regulatory role of endogenous VKDPs on SVZ cell proliferation and identifies the two structurally related factors Gas6 and protein S as the two only secreted VKDPs produced by SVZ cells. While Gas6 promotes stem-like cell proliferation and/or pools maintenance, protein S inhibits SVZ cell proliferation and counterbalances warfarin mitogenic effects. Therefore, the observed mitogenic effect of warfarin on SVZ cells is mainly due to the suppression of endogenous protein S production implying thereby that protein S is an endogenous inhibitor of cell proliferation within the SVZ germinative center. The original phenotype associated with Gas6^−/−^ mice is a defect in platelet aggregation [27]. In the process of this study, we observed in Gas6^−/−^ mice, a significant decrease in both stem cell proliferation or stem cell pool within the SVZ and in neurogenesis in the olfactory bulb associating thereby a novel loss of function phenotype with Gas6^−/−^ mice. Our data showing the production of Gas6 and protein S as well as the expression and activation pattern of their tyrosine kinase receptors TAM together with loss of function experiments suggest that under physiological conditions, Gas6 and protein S regulate SVZ cell growth. A recent study, based on comparative expression profiling between purified neural progenitor cells and their neuronal progeny derived from the developing mouse cerebral cortex, uncovered an important role of Tyro3/Axl/Mer receptor tyrosine kinases in the maintenance of neural progenitor cells during development [43]. In this report, we demonstrate that Tyro3/Axl/Mer receptor tyrosine kinase expression is also expressed by postnatal and adult NSCs, and we establish a functional role of the Tyro3/Axl/Mer ligands, protein S, and Gas6, on cell proliferation, maintenance of the SVZ brain subventricular stem cell niche, and neurogenesis.

The pattern of BrdU-positive cells within and around the SVZ observed following warfarin intracerebroventricular injection resembles that described following EGF injection [44], which suggests that it represents a response to the balance leaning toward proliferation either by the exogenous addition of a mitogen (EGF) or the removal of an endogenous inhibitory block (endogenous protein S). Endogenous protein S inhibitory action for SVZ cell proliferation may prevent neural stem/progenitor cells depletion contributing thereby toward the maintenance of the repair potential of the SVZ. As deregulation of SVZ stem/progenitor cell proliferation can result in the development of brain cancers [45–48], it may also represent a constitutive mechanism for preventing brain tumor formation.

Warfarin or its derivatives are widely used for acute and chronic treatments of thromboembolic disorders among which stroke with a daily dose of 1–10 mg [14, 15]. The evidence we establish for a regulatory role of at least two SVZ endogenous VKDPs, protein S and Gas6, for neural precursors proliferation should be taken into account for the use of warfarin for treating stroke and cerebral ischemia. Warfarin embryopathy, characterized by several central nervous system anomalies, is associated with warfarin exposure in the second or the third trimester in human [49]. The pathogenesis of these brain anomalies are simply ascribed to fetal bleeding events [50]. The fact that warfarin promotes SVZ cell proliferation in vitro together with data showing that its direct intracerebroventricular injection induces cell proliferation in vivo which was mimicked by anti-protein S antibody intracerebroventricular injection warrants analyzing further, at a cellular level potential consequences of such anticoagulant therapies on brain's pathogenesis and its relation to Gas6 and protein S loss of function. The γ-glutamyl carboxylase enzyme is expressed in the neuroepithelium during development and its expression in the brain persists during adulthood [51]. In our study, we observed that SVZ cells express the γ-carboxylase protein, which suggests that locally produced brain VKDP can be γ-carboxylated and could act as autocrine or paracrine factors for the neuroepithelium or for SVZ cells that derive from it [52]. Recently, a redox-mediated regulatory mechanism of primary brain-derived NSC function has been described [53]. Brain progenitors cells with characteristics of NSC maintained a high reactive oxygen species (ROS) status and are highly responsive to ROS stimulation [53]. Interestingly, concomitantly with the γ-carboxylation modification that generates VKDPs, ROS are generated [14–18]. Based on the belief that ROS are cytotoxic, vitamin K or its derivative are used as radiation sensitizer or in combination with chemotherapeutic drugs to treat several types of cancer among which glioma [54, 55]. The recent demonstration for ROS promoting effects on NSC self-renewal and neurogenesis [53] together with the evidence we provide for the regulation of NSC proliferation by the SVZ endogenous VKDP, underline the importance of investigating the vitamin K status within the brain and open up new perspectives for investigating further the biological significance of the expression of the VKDPs, Gas6 and protein S and their tyrosine kinase receptors TAM within the SVZ neurogenic niche or other stem cell systems.

## CONCLUSION

Our study uncovers that the two VKDPs, Gas6 and protein S, are major regulators for cell proliferation and stemness within the SVZ neurogenic niche. Our data open up new perspectives for investigating further the role of vitamin K, VKDPs, and anticoagulants in NSC biology in health and disease.
